# Clinical Features of Surgically Managed Adnexal Masses in Children and Adolescents at a Tertiary Referral Center

**DOI:** 10.3390/children13040549

**Published:** 2026-04-15

**Authors:** Jimin Bae, Hyun Kyung Kim, Inha Lee, SiHyun Cho, Young Sik Choi, Joo Hyun Park

**Affiliations:** 1 Department of Obstetrics and Gynecology, Gangnam Severance Hospital, College of Medicine, Yonsei University, Seoul 06273, Republic of Korea; bgm110099@yuhs.ac (J.B.); ihlee86@yuhs.ac (I.L.); sihyuncho@yuhs.ac (S.C.); 2 Department of Laboratory Medicine, College of Medicine, Yonsei University, Seoul 03722, Republic of Korea; kalmiaria@yuhs.ac; 3 Institute of Women’s Life Medical Science, College of Medicine, Yonsei University, Seoul 03722, Republic of Korea; yschoi08@yuhs.ac; 4 Department of Obstetrics and Gynecology, Severance Hospital, College of Medicine, Yonsei University, Seoul 03722, Republic of Korea; 5 Department of Obstetrics and Gynecology, Yongin Severance Hospital, College of Medicine, Yonsei University, Yongin 16995, Republic of Korea

**Keywords:** adnexal mass, children, adolescents, ovary-sparing surgery, ovarian function preservation, ovarian torsion

## Abstract

**Highlights:**

**What are the main findings?**

- Among 77 surgically managed ovarian and adnexal masses in patients aged 6–18 years, 84.4% were clinically non-malignant, and torsion occurred only in patients aged 15 years or younger.
- All borderline or malignant tumors showed an intermediate or high risk of malignancy on ultrasound (Ovarian-Adnexal Reporting and Data System; O-RADS categories 4–5); fertility-sparing surgery was achieved in 11 of 12 patients, and 11 of 12 had no evidence of disease after a median follow-up of 4 years and 2 months.

**What are the implication of the main findings?**

- Preoperative ultrasonography combined with tumor marker assessment can support practical risk stratification in children and adolescents with adnexal masses.
- Ovarian preservation is feasible in most surgically managed cases, including many borderline or malignant tumors, when treatment is planned with oncologic safety in mind.

**Abstract:**

**Background/Objectives:** We aimed to characterize age-related clinicopathologic features, preoperative risk stratification, surgical management, and menstrual and reproductive outcomes of surgically managed ovarian and adnexal masses in children and adolescents. **Methods:** This retrospective study included 77 patients (aged 6–18 years) treated at a tertiary center between 2010 and 2020. Patients were grouped by age (6–12, 13–15, 16–18 years). **Results:** Abdominal pain (63.6%) was the most common presentation, and laparoscopic surgery was performed in 72.7% of cases. Overall, 84.4% of masses were non-malignant, with mature cystic teratoma being the most common (39.0%). Twelve patients (15.6%) had borderline or malignant tumors. Cyst diameter peaked in the 13–15 year group (*p* = 0.04), while torsion (9.1%) occurred exclusively in patients aged ≤15 years (*p* < 0.01). Preoperative O-RADS accurately stratified risk: all borderline or malignant tumors were O-RADS 4–5, whereas 84.6% of non-malignant lesions were O-RADS 2–3. Elevated CA-125 and AFP correlated well with epithelial and malignant germ cell tumors, respectively. Ovary-sparing surgery (OSS) was achieved in 11 of 12 patients with borderline or malignant tumors; after a median 4.2-year follow-up, 11 were alive without disease, and 10 had resumed menstruation. **Conclusions:** Most pediatric and adolescent adnexal masses are non-malignant. Age influences mass size and torsion risk. Preoperative O-RADS combined with tumor markers effectively aids risk stratification. Fertility preservation is feasible and safe for nearly all patients, supporting conservative surgical planning when intervention is necessary.

## 1. Introduction

Ovarian and adnexal masses are rare entities during childhood and adolescence, with an overall incidence of 2–3 per 100,000 girls younger than 18 years [1,2]. Approximately 10 to 30 percent of surgically managed masses in this age range are malignant [3,4,5]. Although most lesions are benign or non-neoplastic, the diagnosis of an ovarian mass is an extremely concerning issue for patients and families [6]. Preservation of future fertility is therefore a major consideration [7]. Consequently, ovary-sparing surgery (OSS) has become the standard approach for benign lesions in order to preserve lifelong endocrine function and reproductive potential [8]. Even in malignant germ cell tumors, ovarian preservation surgery combined with appropriate adjuvant chemotherapy has shown excellent survival and reproductive outcomes [9,10].

Because ovarian and adnexal masses in children and adolescents are relatively uncommon, clinicians often have limited experience with these conditions [11]. Uninformed overtreatment may lead to significant morbidity and unnecessary compromise of long-term ovarian function [12]. Unnecessary oophorectomy has been associated with adverse sequelae, including premature ovarian insufficiency, low bone density, and cardiovascular disease [8]. Recent studies have also highlighted variability in the surgical management of benign pediatric adnexal masses according to surgeon specialty, with gynecologic surgeons demonstrating higher ovarian preservation rates and fewer complications than pediatric or general surgeons [13].

To prevent unnecessary oophorectomy and safely promote OSS, accurate preoperative risk stratification is essential [8,14,15]. The characteristics of ovarian masses in children and adolescents should not be assumed to mirror those of adults [16,17]. Structured morphological assessment tools, including the Ovarian-Adnexal Reporting and Data System (O-RADS) ultrasound and MRI criteria, have shown favorable diagnostic performance for identifying malignant ovarian masses in pediatric populations [18,19]. Combining imaging findings with age-specific clinical features and tumor markers may help optimize surgical planning and estimate malignant potential before resection [20,21,22].

The objective of this study was to characterize the clinical, radiologic, pathologic, and surgical profiles of children and adolescents who underwent surgery for ovarian and adnexal masses at a tertiary referral center. Because the present study was designed as a surgically managed cohort, lesions managed conservatively were outside the scope of this analysis. We also examined whether preoperative ultrasonographic risk stratification, summarized according to O-RADS categories, together with tumor marker profiles, could support surgical planning in this population.

## 2. Materials and Methods

### 2.1. Study Design and Patient Population

This single-center retrospective study initially identified 98 patients aged 6–18 years who underwent surgery for ovarian or adnexal masses at the Division of Adolescent Gynecology, Department of Obstetrics and Gynecology, Gangnam Severance Hospital, Yonsei University College of Medicine, Seoul, Republic of Korea, between January 2010 and February 2020. Cases were excluded when retrospective O-RADS categorization was not feasible because of incomplete ultrasonographic documentation or when the lesion fell outside the analytic scope of the present study (e.g., O-RADS 0–1). The final analysis therefore included 77 patients with complete clinicopathologic data. The study was intentionally restricted to surgically managed lesions; conservatively managed lesions were outside the scope of the present analysis. Children younger than 6 years were not included because they are managed by the pediatric surgery division within the same medical center. For age-based comparisons, the cohort was divided into three groups: 6–12 years, 13–15 years, and 16–18 years.

### 2.2. Clinical Evaluation and Surgical Management

A comprehensive review of medical records was performed to extract data on preoperative clinical findings, imaging results, surgical characteristics, pathologic diagnoses, postoperative adjuvant therapy, and outcomes.

Preoperative ultrasound findings were retrospectively categorized according to the Ovarian-Adnexal Reporting and Data System (O-RADS) for the present analysis. For descriptive comparisons, lesions were grouped as O-RADS 2–3 versus O-RADS 4–5.

At our institution, lesions with reassuring ultrasonographic features and no clear symptoms were generally managed with observation and interval follow-up ultrasonography. Surgical intervention was considered for persistent or severe pain, suspected torsion, mass effect or compressive symptoms, interval persistence or growth on follow-up imaging, or ultrasonographic findings concerning for malignancy. Symptomatic lesions underwent surgery across the spectrum of O-RADS 2–5 categories, whereas asymptomatic lesions selected for surgery more commonly had sonographic features corresponding to O-RADS 4–5.

All surgical procedures were performed by gynecologic surgeons within the Division of Adolescent Gynecology. During surgery, frozen sections were sent for intraoperative evaluation when conservative, unilateral ovary-sparing surgery (OSS) was performed for suspected early-stage malignant tumors. To avoid discrepancies associated with frozen section reports and over-diagnosis, there were no immediate conversions to ovarian cancer staging during the initial surgery, unless there was evidence that the stage was overtly advanced or preoperative karyotyping indicated high risk for malignancy. To confirm surgical staging, unilateral salpingo-oophorectomy, biopsy of the contralateral ovary, and lymph node sampling were performed. Partial omentectomy was conducted when clinically indicated. Routine systematic lymphadenectomy was not performed in the absence of evidence of nodal disease.

For clinically oriented analyses related to preoperative risk stratification and surgical planning, final diagnoses were grouped as non-malignant lesions or borderline/malignant tumors. Rare diagnoses were tabulated separately in the detailed pathologic summary because of their low frequency and distinct clinicopathologic characteristics.

### 2.3. Postoperative Follow-Up Protocol

According to our departmental clinical practice protocol, patients with non-malignant ovarian masses were advised to undergo annual ultrasonography, supplemented by tumor marker evaluation when necessary. For patients with borderline or malignant tumors, follow-up imaging (abdominopelvic computed tomography [CT] or PET-CT) and tumor marker evaluations were performed at 3- to 12-month intervals, depending on histologic type, tumor grade, disease stage, and the postoperative therapy administered.

### 2.4. Statistical Analysis

All statistical analyses were performed using Prism version 11 (GraphPad Software, Inc., La Jolla, CA, USA). Continuous variables are presented as means ± standard deviations, and categorical variables are expressed as frequencies and percentages. Continuous variables were compared using one-way analysis of variance (ANOVA), and categorical variables were compared using two-sided Fisher’s exact test. For contingency tables larger than 2 × 2, the exact extension implemented in Prism 11 was used. A *p*-value < 0.05 was considered statistically significant.

## 3. Results

### 3.1. Baseline Patient Characteristics

A total of 77 patients were included in the final analysis. The mean age at presentation was 15.4 ± 2.4 years, and the cohort was distributed into the 6–12-year (15.6%), 13–15-year (29.9%), and 16–18-year (54.5%) groups. The majority of patients (84.4%) were postmenarchal. The most common presenting symptom across all age groups was abdominal pain (63.6%), followed by a palpable abdominal mass or increased abdominal girth (18.2%). A small proportion of patients had underlying conditions, including Müllerian anomalies (2.6%) and a sex chromosome aberration (45,X/46,XY mosaicism, 1.3%) (Table 1).

### 3.2. Preoperative Evaluation and Risk Stratification

Preoperative imaging revealed an overall mean cyst diameter of 11.5 ± 8.8 cm. Notably, mass size was significantly larger in the 13–15-year group (15.4 ± 9.6 cm) than in the other age groups (*p* = 0.04). Preoperative ultrasound classified 71.4% of masses as O-RADS 2–3 (low risk) and 28.6% as O-RADS 4–5 (intermediate to high risk). All 12 patients ultimately diagnosed with borderline or malignant tumors were classified as O-RADS 4–5. Conversely, 55 of 65 non-malignant lesions (84.6%) were classified as O-RADS 2–3, whereas 10 (15.4%) were classified as O-RADS 4–5. Among tumor markers, CA-125 was elevated in 33.8% of the total cohort and was the most sensitive marker for borderline or malignant tumors, being elevated in all epithelial tumor cases. Elevated alpha-fetoprotein (AFP) was observed exclusively in malignant germ cell tumors. Three surgically managed cases were pregnancy-related, including two tubal pregnancies and one hemorrhagic corpus luteum complication during pregnancy; all occurred in the 16–18-year group (Table 2).

### 3.3. Surgical Interventions and Ovarian Preservation

A minimally invasive approach was preferred, with laparoscopic surgery successfully performed in 72.7% of cases. Acute torsion of the mass occurred in 7 patients (9.1%); this complication was observed only in patients aged 15 years or younger and was most frequent in the 6–12-year group (25.0%, *p* < 0.01). A strong emphasis was placed on ovary-sparing surgery (OSS). Cyst enucleation was performed in 67.5% of patients, and the rate of OSS increased with age. Among the 77 patients, non–fertility-sparing surgery was required in two patients (2.6%): one underwent hysterectomy with bilateral salpingo-oophorectomy (BSO) for dysgerminoma, and one underwent bilateral salpingo-oophorectomy for gonadoblastoma. Fertility-preserving surgery was performed in the remaining 75 patients (97.4%) (Table 3).

### 3.4. Pathologic Overview

**Figure 1:**
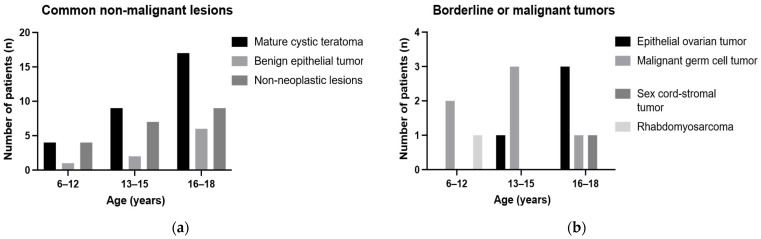
Age-group distribution of selected diagnostic categories. Grouped bar charts showing the number of patients in each diagnostic subgroup across the three age groups (6–12, 13–15, and 16–18 years). (**a**) Common non-malignant lesions, including mature cystic teratoma, benign epithelial tumors, and non-neoplastic lesions. (**b**) Borderline or malignant tumors, including epithelial ovarian tumors, malignant germ cell tumors, sex cord-stromal tumors, and rhabdomyosarcoma. Rare diagnoses were not plotted because of their low frequency and are summarized separately in Table S1.

Histopathologic analysis showed that 65 of 77 lesions (84.4%) were clinically categorized as non-malignant and 12 (15.6%) as borderline or malignant. For descriptive purposes, the 65 non-malignant lesions were further subdivided into 59 common non-malignant diagnoses and 6 rare diagnoses, including gonadoblastoma (*n* = 1) and five miscellaneous diagnoses. The common non-malignant diagnoses consisted of mature cystic teratoma (*n* = 30), benign epithelial tumors (*n* = 9), and non-neoplastic lesions (*n* = 20). Parovarian cysts were diagnosed more frequently in the 6–12-year group than in the older groups (33.3% vs. 13.0% and 4.8%, *p* = 0.02). Borderline or malignant tumors included epithelial ovarian tumors (*n* = 4), malignant germ cell tumors (*n* = 6), one Sertoli–Leydig cell tumor, and one rhabdomyosarcoma (Table S1, Figure 1).

### 3.5. Management and Outcomes of Borderline and Malignant Tumors

Of the 12 patients with borderline or malignant tumors, most presented with stage I disease. Patients with stage IA mucinous cystadenocarcinoma or mucinous borderline tumors underwent fertility-sparing surgery without postoperative adjuvant chemotherapy (POAC). By contrast, malignant germ cell tumors and the Sertoli–Leydig cell tumor were treated with surgery followed by POAC, most commonly bleomycin, etoposide, and cisplatin (BEP). One patient with stage IIIB yolk sac tumor underwent second-look surgery with lymph node sampling, partial omentectomy, and excision of peritoneal lesions, after which the contralateral ovary and uterus were preserved. One patient with dysgerminoma and known 45,X/46,XY mosaicism underwent bilateral gonadal removal with excision of pelvic peritoneal implants and lymph node sampling, and a staged second operation was not required. One patient with stage IV rhabdomyosarcoma discontinued palliative chemotherapy because of disease progression. At a median follow-up of 4 years and 2 months, 11 of the 12 patients with borderline or malignant tumors were alive with no evidence of disease, and 10 had resumed regular menstruation (Table 4).

## 4. Discussion

Ovarian cancer in the pediatric age group is uncommon. According to the North American Association of Central Cancer Registries, out of 67,746 ovarian cancers registered between 1992 and 1997, only 481 were diagnosed in patients aged 15–19 years, and 302 cases were in those below the age of 15 years [2]. Despite its rarity, the potential need for surgical management and the possibility of encountering a rare malignancy make it an extremely worrisome event for both patients and their families.

Because of the limited data on ovarian and adnexal masses in children and adolescents, well-established treatment protocols remain lacking. Achieving an optimal balance between surgical radicality and fertility preservation is challenging and often complicates clinical decision-making. We therefore performed this retrospective analysis of surgically managed primary ovarian and adnexal masses at a tertiary referral center in order to provide evidence supporting surgical strategies based on preoperative diagnosis and prognostic assessment.

A thorough understanding of the age-related diagnostic distribution of these lesions is essential to facilitate accurate diagnosis and avoid unnecessary or overly aggressive treatment. Consistent with previous reports, our cohort showed a clear predominance of clinically non-malignant lesions [17,23,24]. For clinically oriented analyses, 65 of 77 lesions (84.4%) were categorized as non-malignant and 12 (15.6%) as borderline or malignant. Among the non-malignant lesions, mature cystic teratoma, benign epithelial tumors, and non-neoplastic lesions were the most common diagnoses, whereas gonadoblastoma and five additional rare diagnoses were summarized separately because of their low frequency. Parovarian cysts were more frequent in the youngest group, although descriptive age-related variation was also observed across other specific diagnoses. Although the overall distribution of the three top-level pathologic groups did not differ significantly across age groups (overall Fisher’s exact *p* = 0.592), specific diagnoses showed descriptive age-related variation; parovarian cysts were more frequent in the youngest group (*p* = 0.02), whereas borderline or malignant tumors were numerically less common in older adolescents.

Symptoms of ovarian and adnexal masses in this population are non-specific. In line with previous reports [20], abdominal pain (63.6%) and increased abdominal girth (18.2%) were the leading presenting symptoms. Because gynecologic screening is not common, adnexal lesions having undergone surgical intervention were discovered at a large mean diameter of 11.5 ± 8.8 cm overall. Young patients rarely describe the pain specifically as pelvic; vague abdominal discomfort is more common unless acute rupture or torsion has occurred. Surgically confirmed torsion occurred in 9.1% of cases and was observed exclusively in patients aged 15 years or younger. Although all torsion-associated lesions in our study were benign, the apparent torsion rate may have been influenced by selection bias because many asymptomatic cases are managed conservatively. Nevertheless, in younger patients presenting with an ovarian mass and severe cyclic pain, prompt evaluation for torsion and timely surgical intervention are warranted to prevent ovarian necrosis and potential fertility loss.

This selection pattern should be interpreted in light of real-world surgical triage in pediatric adnexal masses. Prior pediatric literature that included both conservatively managed and surgically treated lesions showed that surgically treated masses were larger and more often neoplastic on ultrasonography than lesions managed conservatively. Many observed lesions regressed spontaneously on follow-up, whereas some sonographically non-neoplastic lesions still underwent surgery because severe abdominal pain raised concern for torsion [25]. This pattern is consistent with our institutional practice, in which lesions with reassuring sonographic features and no clear symptoms were usually observed, whereas surgery was considered for persistent or severe pain, suspected torsion, mass effect or compressive symptoms, interval persistence or growth, or sonographic features corresponding to higher O-RADS categories. Other surgically managed pediatric series have likewise reported a predominance of benign ovarian lesions [24].

Because clinical symptoms are unreliable clues, imaging studies and tumor marker evaluations must be interpreted together. In this study, all lesions ultimately diagnosed as borderline or malignant were classified as O-RADS 4–5 on preoperative ultrasound, whereas 84.6% of non-malignant lesions were classified as O-RADS 2–3. These findings support the practical value of O-RADS-based risk stratification in children and adolescents. Regarding tumor markers, CA-125 was most frequently elevated among borderline or malignant epithelial tumors, whereas AFP elevation was restricted to malignant germ cell tumors. Larger multicenter studies will be needed to clarify associations between specific histologic subtypes and tumor marker profiles.

When borderline or malignant ovarian tumors were suspected, surgery was performed for histopathologic confirmation and tumor excision. Frozen-section analysis was actively used when intraoperative findings were indeterminate and ovary-sparing management was being considered, although the decision for comprehensive surgery was deferred until the final pathology report confirmed the diagnosis in order to prevent overdiagnosis. Fertility-sparing surgery was sought in 11 of the 12 patients with borderline or malignant tumors, with the exception of one patient with dysgerminoma diagnosed with known mosaicism of the sex chromosome with a karyotyping of 45,X/46,XY. Whereas previous literature has reported germ cell malignancies as the most frequent subtype among 15–19-year-olds and ovarian carcinoma as a more common subtype in older adolescents and young adults [2,11], malignant germ cell tumors in our cohort were observed mainly in patients aged 15 years or younger, whereas borderline and epithelial ovarian tumors were identified only in adolescents. In our cohort, postoperative adjuvant chemotherapy was reserved mainly for malignant germ cell and sex cord-stromal tumors, whereas stage IA mucinous cystadenocarcinoma and borderline tumors were managed without adjuvant chemotherapy.

Due to the low incidence of pediatric ovarian tumors, our cases were collected over a 10-year period (2010–2020). Despite variable follow-up durations, 11 of the 12 patients with borderline or malignant tumors remain alive with no evidence of disease, and 10 resumed menstruation, with the exception of one with dysgerminoma having undergone hysterectomy with bilateral ovotestis removal. Nonetheless, the long-term impact of chemotherapy on fertility requires ongoing evaluation. Previous studies report fertility impairment in up to 15% of pediatric cancer survivors, particularly those treated with alkylating agents [6]. Gonadotropin-releasing hormone analog administration prior to chemotherapy has been reported to offer protective effects on ovarian function [7]. In our cohort, leuprolide acetate was administered to 7 of the 8 patients undergoing chemotherapy, approximately two to three weeks before treatment initiation.

This study has several limitations. First, because the cohort was recruited from a single tertiary referral center, the histologic composition of surgically managed ovarian and adnexal masses may differ from that of the general pediatric and adolescent population. Second, the retrospective design entails inherent biases, including a relatively small sample size and a broad timeline of case inclusion, which may limit generalizability. No a priori power calculation was performed because this was a retrospective series of a rare condition that included all eligible surgically managed cases during the study period. Third, because this study was restricted to surgically managed cases, the cohort overrepresents symptomatic, persistent, or radiologically concerning lesions and does not reflect the full spectrum of pediatric adnexal masses managed conservatively. Accordingly, our findings are most generalizable to children and adolescents selected for surgery at a tertiary referral center rather than to all pediatric adnexal masses. We did not include a non-surgical comparison cohort because the primary objective of this study was to correlate preoperative imaging-based risk stratification with definitive histopathologic diagnosis in resected lesions; inclusion of observed lesions without pathologic confirmation would have introduced verification bias.

## 5. Conclusions

Most surgically managed ovarian and adnexal masses in this cohort were non-malignant. Preoperative O-RADS assessment combined with tumor marker evaluation may support practical risk stratification and surgical planning in children and adolescents. When surgery is required, fertility-sparing management should remain a central priority whenever oncologically feasible.

## Figures and Tables

**Table 1:** Patient characteristics.

Parameters	Value (N = 77)
**Age (years), *n* (%)**	
Mean ± SD (95% CI)	15.4 ± 2.4 (14.9–16.0)
6–12 years	12 (15.6)
13–15 years	23 (29.9)
16–18 years	42 (54.5)
**BMI (kg/m^2^)**	
Mean ± SD (95% CI)	21.1 ± 3.5 (20.3–21.9)
**Menarche**	
Postmenarchal, n (%)	65 (84.4)
Age at menarche (years), mean ± SD (95% CI)	12.6 ± 1.3 (12.3–12.9)
**Presenting Symptoms, *n* (%)**	
Abdominal pain	49 (63.6)
Palpable abdominal mass/increased girth	14 (18.2)
Menstrual irregularity	8 (10.4)
Amenorrhea	4 (5.2)
Bowel symptoms	2 (2.6)
**Underlying conditions, *n* (%)**	
None	71 (92.2) ^1^
Chromosomal aberration	1 (1.3) ^2^
Müllerian anomaly	2 (2.6)
Mitochondrial disease	2 (2.6)
Precocious puberty	1 (1.3)

^1^ In one patient with gonadoblastoma, the suspected chromosome aberration could not be defined and karyotyping was performed at another center before referral, ^2^ 45,X/46,XYmosaicism. Abbreviations: SD = standard deviation; BMI = body mass index.

**Table 2:** Parameters of preoperative evaluation.

Parameters	Total (N = 77)	6–12 yrs (*n* = 12)	13–15 yrs (*n* = 23)	16–18 yrs (*n* = 42)	*p*-Value
**Hemoglobin, Mean ± SD (g/dL)**	12.5 ± 1.6	13.0 ± 1.3	12.6 ± 1.4	12.3 ± 1.8	0.36
**Diagnostic imaging**					
Cyst diameter, mean ± SD (cm)	11.5 ± 8.8	9.5 ± 6.8	15.4 ± 9.6	9.9 ± 8.3	0.04
O-RADS ^1^, *n* (%)					0.06
2–3	55 (71.4)	5 (41.7)	18 (78.3)	32 (76.2)	
4–5	22 (28.6)	7 (58.3)	5 (21.7)	10 (23.8)	
**Elevated tumor marker rates** **, *n* (%)**					
CA-125	26 (33.8)	4 (33.3)	10 (43.5)	12 (28.6)	0.48
CA-19-9	20 (26.0)	3 (25.0)	7 (30.4)	10 (23.8)	0.84
AFP	6 (7.8)	2 (16.7)	2 (8.7)	2 (4.8)	0.58
Pregnancy-related ^2^, n (%)	3 (3.9)	0 (0)	0 (0)	3 (7.1)	0.15

^1^ Patients with O-RADS 1 lesions were generally managed non-surgically and therefore were not represented in this surgically managed cohort. O-RADS 2–3 indicates a low risk of malignancy, and O-RADS 4–5 indicates an intermediate to high risk. The *p*-value was calculated for the overall distribution of O-RADS categories across the three age groups using the exact extension of Fisher’s exact test. ^2^ Includes two tubal pregnancies and one hemorrhagic corpus luteum complication during pregnancy. Abbreviations: SD = standard deviation; AFP = alpha-fetoprotein.

**Table 3:** Intraoperative parameters.

Parameters	Total (N = 77)	6–12 yrs (*n* = 12)	13–15 yrs (*n* = 23)	16–18 yrs (*n* = 42)	*p*-Value
Torsion of mass, *n* (%) ^1^	7 (9.1)	3 (25.0)	4 (17.4)	0 (0)	<0.01
**Mode of su** **r** **gery, *n* (%)**					0.19
Laparoscopy	56 (72.7)	7 (58.3)	15 (65.2)	34 (81.0)	
Laparotomy	21 (27.3)	5 (41.7)	8 (34.8)	8 (19.0)	
**Type of surgery** **, *n* (%)**					
Cyst enucleation	52 (67.5)	5 (41.7)	15 (65.2)	32 (76.2)	0.07
Salpingo-oophorectomy ^2^	24 (31.2)	7 (58.3)	7 (30.4)	10 (23.8)	0.07
Hysterectomy with BSO	1 (1.3)	0 (0)	1 (4.3)	0 (0)	0.45

^1^ Includes hemorrhagic corpus luteal cysts (n = 4), mature cystic teratoma (n = 2), serous cystadenoma (n = 1). ^2^ Includes unilateral salpingo-oophorectomy (USO), bilateral salpingo-oophorectomy (BSO), and USO with contralateral ovarian biopsy plus lymph node sampling. Abbreviations: BSO = bilateral salpingo-oophorectomy.

**Table 4:** Stage, postoperative adjuvant chemotherapy, and outcomes of patients with borderline or malignant tumors in the cohort.

Tumor Pathology	Stage	Chemotherapy Regimen	Follow-Up Period/ Prognosis
Mucinous cystadenocarcinoma	IA	None	2 yr 5 mo (NED)
	IA	None	2 yr (NED)
Mucinous borderline tumor	IA	None	7 yr 10 mo (NED)
	IA	None	1 yr 4 mo (NED)
Yolk sac tumor	IA	BEP	1 yr (NED)
	IIIB	BEP	5 yr 2 mo (NED)
Mixed germ cell tumor	IA	BEP	8 yr 3 mo (NED)
Dysgerminoma	IIA	BEP	7 yr 8 mo (NED)
Immature teratoma	IA	BEP	4 yr 2 mo (NED)
	IA	Bleomycin	2 yr (NED)
Sertoli-Leydig cell tumor	IA	BEP	4 yr (NED)
Rhabdomyosarcoma	IV	Cisplatin ^1^/ Gemcitabine ^2^	Cessation of treatment (Disease progression)

^1^ Neoadjuvant chemotherapy. ^2^ Palliative chemotherapy. Abbreviations: BEP = bleomycin, etoposide, cisplatin; NED = no evidence of disease.

## Data Availability

The data presented in this study are available on request from the corresponding author. The data are not publicly available due to privacy and ethical restrictions.

## Supplementary Materials

The following supporting information can be downloaded at: https://www.mdpi.com/article/10.3390/children13040549/s1, Table S1: Final pathologic diagnoses according to age group.

## Abbreviations

The following abbreviations are used in this manuscript:

OSS	Ovary-sparing surgery
O-RADS	Ovarian-Adnexal Reporting and Data System
POAC	Postoperative adjuvant chemotherapy
BEP	Bleomycin, etoposide, and cisplatin
CT	Computerized tomography
PET-CT	Positron emission tomography-computed tomography
NED	No evidence of disease
