# Supplementary material for: Clinical Features of Surgically Managed Adnexal Masses in Children and Adolescents at a Tertiary Referral Center
Source: Children (Basel). 2026 Apr 15;13(4):549. doi: 10.3390/children13040549 (PMC13114405; doi:10.3390/children13040549)
Supplement: Supplementary file 1 [file children-13-00549-s001.zip › children-4249201-supplementary.pdf]

Table S1. Final pathologic diagnoses according to age group

Pathologic diagnosis	Total (N = 77), n(%)	6–12 yrs (n = 12) , n(%)	13–15 yrs (n = 23) , n(%)	16–18 yrs (n = 42) , n(%)	p-value
**Non-malignant lesions†**	**59 (76.6)**	**9 (75.0)**	**18 (78.3)**	**32 (76.2)**	**1.00**
**Benign germ cell tumor**	**30 (39.0)**	**4 (33.3)**	**9 (39.1)**	**17 (40.5)**	0.95
Mature cystic teratoma	30 (39.0)	4 (33.3)	9 (39.1)	17 (40.5)	
**Benign epithelial tumor**	**9 (11.7)**	**1 (8.3)**	**2 (8.7)**	**6 (14.3)**	**0.89**
Mucinous cystadenoma	4 (5.2)	0 (0)	2 (8.7)	2 (4.8)	
Serous cystadenoma	4 (5.2)	1 (8.3)	0 (0)	3 (7.1)	
Endometriotic cyst	1 (1.3)	0 (0)	0 (0)	1 (2.4)	
**Non-neoplastic lesions**	**20 (26.0)**	**4 (33.3)**	**7 (30.4)**	**9 (21.4)**	**0.59**
Parovarian cyst	9 (11.7)	4 (33.3)	3 (13.0)	2 (4.8)	0.02
Hemorrhagic corpus luteal cyst	10 (13.0)	0 (0)	4 (17.4)	6 (14.3)	0.42
Tubo-ovarian abscess	1 (1.3)	0 (0)	0 (0)	1 (2.4)	1.00
**Rare diagnoses†**	**6 (7.8)**	**0 (0)**	**1 (4.3)**	**5 (11.9)**	**0.52**
Gonadoblastoma	1 (1.3)	0 (0)	0 (0)	1 (2.4)	
Others	5 (6.5)	0 (0)	1 (4.3)	4 (9.5)	
**Borderline or malignant tumors†**	**12 (15.6)**	**3 (25.0)**	**4 (17.4)**	**5 (11.9)**	**0.47**
**Epithelial ovarian tumor**	**4 (5.2)**	**0 (0)**	**1 (4.3)**	**3 (7.1)**	**1.00**
Mucinous cystadenocarcinoma	2 (2.6)	0 (0)	0 (0)	2 (4.8)	
Mucinous borderline tumor	2 (2.6)	0 (0)	1 (4.3)	1 (2.4)	
**Malignant germ cell tumor**	**6 (7.8)**	**2 (16.7)**	**3 (13.0)**	**1 (2.4)**	**0.10**
Yolk sac tumor	2 (2.6)	0 (0)	1 (4.3)	1 (2.4)	
Mixed germ cell tumor	1 (1.3)	0 (0)	1 (4.3)	0 (0)	
Dysgerminoma	1 (1.3)	0 (0)	1 (4.3)	0 (0)	
Immature teratoma	2 (2.6)	2 (16.7)	0 (0)	0 (0)	
**Sex cord-stromal tumor**	**1 (1.3)**	**0 (0)**	**0 (0)**	**1 (2.4)**	**1.00**
Sertoli-Leydig cell tumor	1 (1.3)	0 (0)	0 (0)	1 (2.4)	
**Rhabdomyosarcoma**	**1 (1.3)**	**1 (8.3)**	**0 (0)**	**0 (0)**	**0.16**

For clinically oriented analyses, gonadoblastoma and miscellaneous rare diagnoses were included within the overall non-malignant category. In this detailed pathologic summary, these uncommon entities were displayed separately. Row-specific p-values are shown in the last column. † Overall comparison of the three top-level pathologic groups across age groups by Fisher's exact test: $p = 0.592$. "Others" comprised.
